# Intermittent ketogenic fasting with medium-chain triglycerides improves ataxia in COQ8A-related coenzyme Q10 deficiency: A case report

**DOI:** 10.1016/j.ymgmr.2026.101296

**Published:** 2026-02-12

**Authors:** Wiebke Hahn, Karla Erffmeier, Maximilian Schulze, Felix Zahnert, Susanne Knake, Panagiota-Eleni Tsalouchidou

**Affiliations:** a Department of Neurology, Philipps-University Marburg, Baldingerstraße, 35043 Marburg, Germany; b Epilepsy Center Hessen, Department of Neurology, Philipps University Marburg, Marburg, Germany; c Department of Neuroradiology, Philipps-University Marburg, Baldingerstraße, 35043 Marburg, Germany; d Second Department of Neurology, Attikon University Hospital, National and Kapodistrian University of Athens, Greece

**Keywords:** Mitochondrial diseasse, Coenzyme Q10 deficiency, Ataxia, IF-MCT study, Ketogenic diet, Intermittent fasting

## Abstract

**Background:**

Mutations in COQ8A cause primary coenzyme Q10 deficiency, which can present clinically heterogeneously: Symptoms range from cerebellar ataxia, epilepsy, encephalomyopathy, macular degeneration to nephropathy. High-dose coenzyme Q10 supplementation is widely used, yet there is little evidence on complementary strategies, particularly for non-epileptic features such as cerebellar ataxia.

**Case presentation:**

We report a 46-year-old female with genetically confirmed COQ8A-related coenzyme Q10 (CoQ10) deficiency, presenting with ataxia and epilepsy characterized by myoclonic and bilateral tonic–clonic seizures, who participated in a clinical protocol of ketogenic intermittent fasting, a method of intermittent fasting combined with medium-chain triglycerides (MCT) primarily designed for seizure management. The patient followed a 16:8 intermittent fasting regime combined with MCT intake for three months, followed by three months of all-alone intermittent fasting. Routine blood markers and brain MRI, including diffusion imaging were obtained before and after ketogenic fasting.

**Results:**

During the study protocol, while no seizure reduction in myoclonic seizures could be observed, ataxia - quantified by the Scale for the Assessment and Rating of Ataxia (SARA) - improved significantly from 8.5 to 6.0 during the interventions. MRI showed a trend suggesting improved cerebellar microstructural integrity.

**Conclusions:**

This case highlights the potential of ketogenic intermittent fasting as an adjunct therapy for mitochondrial ataxia. Ketogenic intermittent fasting was associated with clinically meaningful improvement of ataxia in a patient with COQ8A-related CoQ10 deficiency, suggesting that ketogenic dietary strategies may represent a promising adjunct therapeutic approach for mitochondrial ataxia. Future research should assess this intervention in larger patient cohorts to confirm its potential benefits.

## 1 Background

Ubiquinone, also known as coenzyme Q10 (CoQ10), is a lipid with a hydrophobic side chain composed of ten isoprene units. It functions as an electron carrier transferring electrons from respiratory chain complexes I and II to complex III and is additionally involved in fatty acid β-oxidation and pyrimidine biosynthesis [1]. Although more than 15 genes have been implicated in CoQ10 biosynthesis—including PDSS1, PDSS2, COQ2, COQ4, COQ6, COQ8, and COQ9—the biosynthetic pathway is not yet fully understood.

Pathogenic variants in these genes cause primary CoQ10 deficiency, which is clinically heterogeneous and may present with cerebellar ataxia, epilepsy, encephalomyopathy, macular degeneration, or nephropathy. Mutations in ADCK3 (COQ8A) are frequently associated with epilepsy and progressive cerebellar ataxia [2,3]. While high-dose CoQ10 supplementation can improve clinical outcomes, therapeutic responses are often incomplete, and treatment options remain limited.

The ketogenic diet (KD) is an established adjunctive therapy for drug-resistant epilepsy and represents the treatment of choice for glucose transporter type 1 (Glut1) deficiency syndrome and pyruvate dehydrogenase deficiency. It is also recommended for mitochondrial disorders affecting respiratory chain complex I [4].

The KD is a high-fat, low-carbohydrate diet with adequate protein intake that induces a fasting-like metabolic state, characterized by hepatic ketone body production, predominantly β-hydroxybutyrate. Proposed mechanisms include modulation of neurotransmitter balance, anti-inflammatory effects, interactions with the gut microbiome, and reduced oxidative stress, potentially mediated by decreased nicotinamide adenine dinucleotide phosphate (NADPH) oxidase activity [4,5]. Due to limited adherence and tolerability, particularly in adults, alternative dietary approaches such as the classical KD, modified Atkins diet (MAD), medium-chain triglyceride (MCT) diet, and low glycemic index treatment have been developed [4,6].

Evidence for the efficacy of ketogenic interventions in mitochondrial diseases is strongest in epilepsy-associated phenotypes, particularly when mutations affect complex I or enzymes of the citric acid cycle [7]. In a systematic review, Zweers et al. [8] reported 20 predominantly pediatric cases with genetically heterogeneous mitochondriopathies treated with classical KD or MAD, with improvement of seizures and, in some cases, motor symptoms [8]. However, data on adult patients remain scarce. Here, we report a 46-year-old woman with genetically confirmed COQ8A-related CoQ10 deficiency presenting with ataxia and epilepsy, who underwent a clinical protocol combining intermittent fasting with medium-chain triglyceride supplementation [9].

## 2 Case report

### 2.1 Patient's history

We report a 46-year-old right-handed female with genetically confirmed COQ8A-related primary coenzyme Q10 deficiency. Since the age of 13, there has been a slowing of motor skills and coordination, especially in the upper extremities. There was also a visual impairment with a narrowing of the visual field. At the age of 26, the patient experienced her first bilateral tonic-clonic seizures, and antiseizure medication (ASM) with carbamazepine was initiated. In 2021, bilateral tonic-clonic seizures increased from a frequency of once every two years to once monthly, resulting in status epilepticus. Seizure control was achieved by adding lacosamide, brivaracetam, and stiripentol to the existing ASM regimen (for details see [10]). Given that the parents were known to be consanguineous and the patient's sister exhibited a similar clinical course, both sisters underwent genetic testing. This confirmed an COQ8A-associated primary coenzyme Q10 deficiency in both. As a result, the patient's treatment regimen was supplemented with high-dose oral coenzyme Q10 (3000 mg/d).

At her reassessment in October 2023, she remained free of bilateral tonic–clonic seizures on adjusted antiseizure medication and CoQ10 supplementation but continued to experience continuous myoclonic seizures with a frequency of several episodes per month, along with persistent visual impairment from retinitis pigmentosa and both gait and upper-limb ataxia.

Due to persistent drug-resistant myoclonic seizures, the patient was enrolled in a clinical study investigating ketogenic intermittent fasting as a combination of intermittent fasting with medium-chain triglycerides (IF-MCT) in patients with drug resistant epilepsy [9].

### 2.2 IF-MCT study and study participation

The prospective two-armed IF-MCT study investigates the effect of intermittent fasting using the 16:8 method combined with a once-daily intake of certified medium-chain fatty acids on seizure frequency in patients with drug-resistant epilepsy [9]. During the IF-MCT study, participants are fasting for three months using the 16:8 method during the first study phase. In the second study phase - at the end of the 16-h fasting phase- patients are drinking a certified dietary supplement with medium-chain fatty acids^1^ with a ketogenic ratio of 2:1. During the first study phase, study visits are planned at three times.^2^ After a one-month break without any restrictions regarding fasting, the second study phase begins. During the second study phase, three study visits are planned.^3^ Due to the within-subject design, each participant receives each study condition. The study protocol was approved by the local ethics committee (AZ-23/92 BO) and registered at ClinicalTrials.gov [9]. During the entire study period, daily seizures were documented using a seizure diary and daily eating and fasting phases were documented using a daily food diary.

In the first phase of the study, the patient was randomized to start with the intermittent fasting plus MCT condition. Throughout the intervention, her baseline medication remained unchanged: coenzyme Q10 (1000 mg 1-1-1), brivaracetam (200 mg 1-0-1), clobazam (5 mg 1-0-1), stiripentol (250 mg 1-0-1), carbamazepine (400 mg 1-0-1) as well as vitamin B1 (250mg 1-0-0), vitamin C (500 mg 1-0-1), and levocarnitine (1000 mg 1-0-1). The patient gave her informed consent to participate in the study.

### 2.3 Methods

#### 2.3.1 Clinical evaluation

To record the frequency of seizures, the patient kept a daily seizure diary. To objectify ataxia, SARA score [11] was recorded by the same study doctor before and after the intervention. The additional recording of SARA score was the only deviation from the IF-MCT protocol.

#### 2.3.2 Laboratory monitoring

Following the study protocol, regular blood counts were carried out to monitor LDL cholesterol levels at each study visit. To monitor the development of any arteriosclerosis during the intervention period, a duplex sonographic examination of the intima media thickness (Siemens S-1000, linear transducer) of common carotid artery (ACC; 1 cm proximal to the bulb) on both sides was carried out at T0.1, T1.2 and T2.2. Furthermore, the patient was asked to measure and document beta-hydroxybutyrate concentration (BHB) in the blood once a week at the end of the fasting episode and 30 min after drinking the MCT drink respectively eating.

#### 2.3.3 Neuroimaging

To assess potential neuroanatomical and microstructural changes associated with the intervention, magnetic resonance imaging (MRI) was performed at T0.1, at the end of the first intervention phase (T1.2), and at the end of the second phase (T2.2). All scans were acquired using the same 3 Tesla Siemens Trio scanner (Siemens Healthcare, Erlangen, Germany) to ensure consistency across measurements.

#### 2.3.4 Structural MRI acquisition

A cranial magnetic resonance imaging with diffusion weighting was performed at three measurements to demonstrate neuronal connectivity during the intervention. Pre- and post-KD 3D-T1-weighed scans (MPRAGE) were acquired with 1 mm isovoxel resolution, TR = 1.9 ms, TI = 0.9 s, Flip angle = 9°, TE = 2.26 and 2.52 ms. At both timepoints, diffusion MRI scans were acquired with an EPI sequence at 2x2x2.4 mm resolution, 30 gradient directions, b-value = 1000, one b0 image, TE = 104 ms, TR = 10.7 s, Flip angle = 90°.

#### 2.3.5 Image processing and metric extraction

Structural T1 images were processed via the recon-all pipeline as implemented in Freesurfer version 7.4.1. This included surface reconstruction, brain extraction, parcellation and segmentation of cortical and subcortical subregions and b0 correction [12]. Registration of structural and diffusion scans was conducted using a boundary-based approach [13]. Given that temporal lobe epilepsy represents the most common form of epilepsy in adults, a control cohort comprising 63 patients with temporal lobe epilepsy was included. This cohort was chosen to account for epilepsy as a common potential confounder, while excluding patients with a mitochondrial or other metabolic etiology of epilepsy. None of these patients exhibited clinical or MRI-detectable morphological evidence of cerebellar involvement. Controls had been scanned on the same 3 T Trio scanner as the patient. Fifty-one controls had been scanned with the same diffusion sequence as the patient. The control cohort, including clinical data and exact diffusion sequences, has already been reported elsewhere [14]. A single-shell implementation of the NODDI model was used to probe cerebellar microstructure in the context of limited diffusion data availability [15]. Cerebellar intra-neurite compartment volume fraction (ICVF) metrics were extracted to probe white matter integrity (for details see Supplementary Table S1). *Z*-scoring of patient pre- and post-KD ICVF metrics was conducted using the mean and standard deviation of ICVF metrics in the control group. Visualization of data was conducted using the seaborn library.

### 2.4 Results

#### 2.4.1 Clinical course during and after the diet

**Fig. 1:**
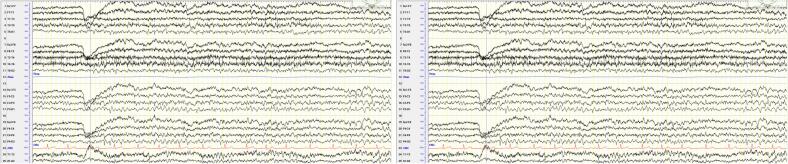
EEG findings of the patient at study start. Montage: longitudinal bipolar. Trace duration: 15 s.

In both clinical phases, the patient remained seizure-free for bilateral tonic-clonic seizures. Regarding myoclonus, there was no improvement in seizure frequency during the intervention phases. The EEG showed normal findings throughout the study period (see Fig. 1).

Regarding ataxia, the average SARA score of the patients at baseline was 8.5 points, which was mainly due to pronounced dysmetria in the *finger chase* and *nose finger tests*. After three months of intermittent fasting with once-daily intake of MCT in the first study phase, the SARA score decreased to 6, mainly due to improved ataxia of the upper extremities. After a one-month break between study phases and after three months of intermittent fasting in the second study phase, the SARA score remained at 6 points, which was mainly due to a persistent improvement in the ataxia in the upper extremities. The measurements of LDL, HDL, triglycerides and total cholesterol did not show any significant changes during the intervention (e.g., LDL concentration at T0.1: 170 mg/dl compared to LDL at T1.2: 169 mg/dl), nor did the intima media thickness measured sonographically (for details see Table 1).

**Table 1:** Low-density lipoprotein (LDL), high-density lipoprotein (HDL), triglycerides, total cholesterol concentration and intima media thickness (IMT) during the study period. IF = intermittent fasting, IMT = intima media thickness, ACC = A. carotis communis.

	Study phase 1: IF + shake			Study phase 2: IF		
	T0.1	T1.1	T1.2	T0.2	T2.1	T2.2
LDL (mg/dl)	170	144	169	152	170	152
HDL (mg/dl)	84	76	83	84	74	82
Triglycerides (mg/dl)	103	128	105	154	166	97
Total cholesterol (mg/dl)	267	245	276	269	262	255
IMT ACC right (mm)	0,4	–	0,4	–	–	0,3
IMT ACC left (mm)	0,4	–	0,5	–	–	0,4

The highest BHB concentration was 0.7 mmol/l 30 min after taking the MCT drink compared to the maximum BHB concentration of 0.2 mmol/l after consuming breakfast (for details see Table 2).

**Table 2:** The concentration of beta-hydroxbutyrate (mmol/l) from the blood of the fingertip measured weekly by the patient. BHB = betahydroxybutyrate, IF = intermittent fasting, m = mean, SD = standard deviation.

	Study phase 1: IF + shake		Study phase 2: IF	
	BHB-concentration at the end of 16 h fasting	BHB-concentration 30 min after shake	BHB-concentration at the end of 16 h fasting	BHB concentration 30 min after eating
Week 1	0.1	0.8	0.1	0.1
Week 2	0.0	0.7	0.1	0.1
Week 3	0.0	*not measured*	0.1	0.1
Week 4	0.0	0.2	0.1	0.1
Week 5	0.0	0.5	0.0	0.1
Week 6	0.1	0.4	0.1	0.2
Week 7	0.1	0.4	0.0	0.1
Week 8	0.1	0.6	0.1	0.1
Week 9	0.1	0.2	0.1	0.1
Week 10	0.1	0.1	0.1	0.2
Week 11	0.0	0.1	0.1	0.0
Week 12	0.1	0.1	0.1	0.1
*m*	*0.05*	*0.37*	*0.08*	*0.10*
*SD*	*0.05*	*0.25*	*0.03*	*0.05*

#### 2.4.2 Imaging outcomes of comparison with TLE-cohort

**Fig. 2:**
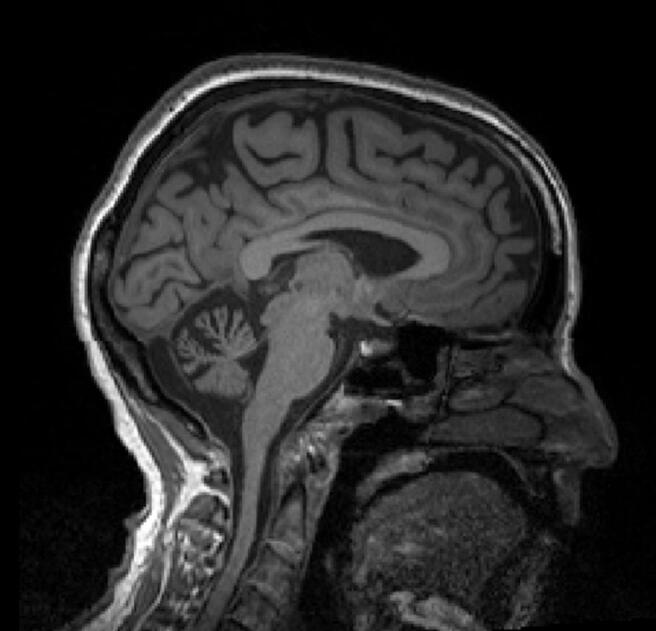
Sagittal cMRI (FLAIR sequence) as an example at measurement time T0.1.

**Fig. 3:**
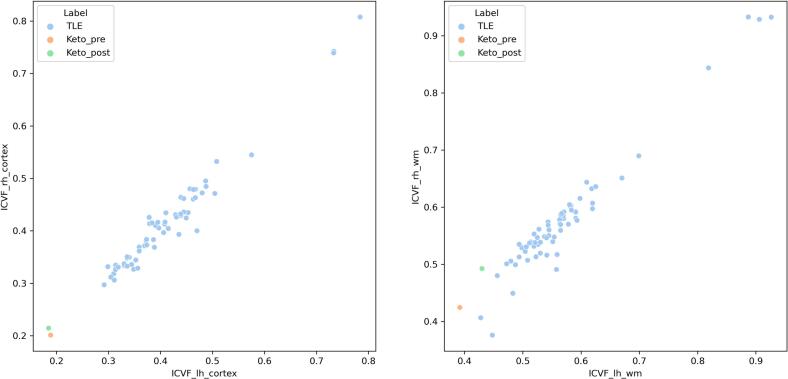
*Z*-scores in relation to TLE-cohort. Improvement after ketogenic diet in ICVF (intra-neurite compartment volume fraction).

MR morphologically, the FLAIR sequence revealed known cerebellar atrophy, which remained unchanged over the study period (see Fig. 2):

After KD, cerebellar white matter (wm) ICVF z-scores improved by 0.4 and 0.7 standard deviations relative to the TLE-control mean for the left (lh) and right hemispheres (rh), respectively (ICVF_lh_wm_pre KD vs. post KD = −1.8, −1.4; ICVF_rh_wm_pre / post = −1.5, −0.8; ICVF_lh_cortex pre / post = −2.1, −2.2; ICVF_rh_cortex pre / post = −2.1, −1.9) (see Fig. 3):

## 3 Discussion

In a within-subject design, a 46-year-old female patient suffering from COQ8A-related coenzyme Q10 deficiency underwent intermittent ketogenic fasting as part of the IF-MCT study [9]. While mild ketosis was achieved, blood lipid levels and intima media thickness remained stable. While no reduction in myoclonic seizures could be observed, there was an improvement in ataxia through ketogenic intermittent fasting, which continued into the study phase with intermittent fasting alone. More specifically, after three months of ketogenic intermittent fasting, the SARA score was reduced from 8.5 to 6 points. This change exceeds the commonly cited threshold of one point for a clinically meaningful difference on the SARA scale, as supported by both anchor-based and distribution-based analyses in ataxia cohorts [16].

No reduction in myoclonus was observed, potentially due to insufficient ketosis, as β-hydroxybutyrate levels remained below the ketogenic range despite an increase following MCT supplementation. β-hydroxybutyrate concentrations are not consistently associated with seizure reduction in drug-resistant epilepsy [17,18]. The sustained improvement in upper extremity ataxia could indicate a possible MCT-induced effect. Medium-chain fatty acids have been shown to exert antiseizure properties via AMPA receptor inhibition and enhanced mitochondrial function [19]. Given that higher MCT doses were used in previous studies, a dose-dependent seizure-suppressive effect cannot be excluded [20].

When looking at the previous literature on the influence of the ketogenic diet on ataxia in mitochondrial diseases, the evidence is limited, primarily due to the heterogeneity of the group of mitochondrial diseases. In a prospective case-control study, Wesól-Kucharska et al. investigated pediatric patients with mitochondrial disease including one patient with COQ8A-mutation on ketogenic regimens and reported significant seizure reductions, with some patients also showing improvement in motor symptoms such as ataxia, tremor, and dystonia, though these changes did not reach statistical significance [21].

Neuroimaging findings in our patient support a potential treatment-related trend. Right hemispheric white matter ICVF increased from −1.46 to −0.79, suggesting improved microstructural integrity. ICVF, derived from diffusion-weighted MRI, reflects neurite density and has been proposed as a marker of cellular integrity in neurodegenerative diseases. Reduced oxidative stress in hippocampal neurons has been proposed as a potential mechanism of the ketogenic diet [5]. Similar effects may occur in the cerebellum, potentially explaining the improvement in ICVF [22]. The findings point to early structural effects of metabolic interventions, which require confirmation in longer-term studies including a healthy control cohort.

### 3.1 Limitations

This report is limited by its single-patient design, which precludes statistical inference and generalizability. Additionally, the absence of blinding, a placebo arm, or control conditions limits the ability to isolate the effects of MCT supplementation versus intermittent fasting alone. The utilized biophysical model [23], does not model cerebrospinal fluid (CSF). While it is well suited for the present analysis of cerebral white matter, cortical analyses are prone to CSF contamination and should be treated with caution.

## 4 Conclusion and significance

To our knowledge, this is the first case study reporting improvement in ataxia in a patient with a COQ8A mutation following a ketogenic intervention. While the precise mechanism remains unclear, neuroprotective effects such as reduced oxidative stress potentially contribute to improved ICVF values [22] and functional outcomes such as improvement of ataxia. These preliminary findings suggest that ketogenic dietary strategies-usually used for epilepsy treatment may hold therapeutic potential also for ataxia in mitochondrial disorders such as COQ8A. Further studies with larger cohorts are necessary to determine the spectrum of potential beneficial influences of the ketogenic diet or its variations on mitochondrial diseases.

## CRediT authorship contribution statement

**Wiebke Hahn:** Writing – original draft, Conceptualization. **Karla Erffmeier:** Writing – review & editing. **Maximilian Schulze:** Writing – review & editing. **Felix Zahnert:** Writing – review & editing. **Susanne Knake:** Writing – original draft. **Panagiota-Eleni Tsalouchidou:** Conceptualization, Writing – original draft, Writing – review & editing.

## Ethics approval and consent to participate

We confirm that we have read the Journal's position on issues involved in ethical publication and affirm that this report is consistent with those guidelines.

## Funding

The IF-MCT study, in which the patient presented took part, was made possible by a donation from the Otto Loewi Foundation, a non-profit organization that promotes research, therapy and training in neurology.

## Declaration of competing interest

Wiebke Hahn has received research grant from the Otto-Loewi foundation as well as honoraria from Kanso, both of which are unrelated to the presented work.

Karla Erffmeier has no conflicts of interest to declare.

Felix Zahnert received funding from the Otfrid Foerster Grant of the German chapter of the ILAE (DGfE), the Clinician Scientist Program (SUCCESS) of the University of Marburg and the LOEWE Research Cluster ADMIT (Advanced Medical Physics in Imaging and Therapy) none of which are unrelated to the presented work.

Maximilian Schulze has no conflicts of interest with respect to this manuscript.

Susanne Knake is managing director of the DGfE, she is a member of the DGKN and in the epilepsy expert panel of the DGN and the EAN. She is playing a leading role in the creation of DGN guidelines on epilepsy and first epileptic seizures as well as status epilepticus. SK is a member of the advisory board of the Epilepsy Foundation of Diakonie Hessen and the Jyette and Peter Wolf Foundation. She has received honoraria for lectures from Bial, Eisai, Desitin, Jazz, Merck Serono and UCB none of which are related to the present work.

Panagiota-Eleni Tsalouchidou has received research grants from the German Society for Epileptology (Otfrid-Foerster Stipendium, DGfE), as well as travel grants and honoraria for lectures from UCB and Angelini none of which are related to the presented work.

## Data Availability

The data that support the findings of this study are available from the corresponding author upon reasonable request.

## Appendix A Supplementary data

Supplementary material: Table S1 is the supplementary data. Image 1

## Footnotes

1 Product: “Kanso KetoEpi 2:1”. Product information: 200 ml per bottle with 360 kcal, 32 g fat (of wich 26 g contains MCT with a ratio of C8: C10 of 50:50), 14.2 g proteins.

2 Study visits take place at the beginning of the first study phase (T0.1), after four weeks (T1.1) and after 12 weeks in the first study phase (T1.2).

3 Study visits take place at the beginning of the second study phase (T0.2), after four weeks (T2.1) and after 12 weeks in the second study phase (T2.2).
