# Supplementary material for: Intermittent ketogenic fasting with medium-chain triglycerides improves ataxia in COQ8A-related coenzyme Q10 deficiency: A case report
Source: Mol Genet Metab Rep. 2026 Feb 12;46:101296. doi: 10.1016/j.ymgmr.2026.101296 (PMC12914829; doi:10.1016/j.ymgmr.2026.101296)
Supplement: Supplementary file 1 — Supplementary material: Table S1 is the supplementary data. [file mmc1.docx]

**Supplementary material:**

| **ODI_lh_cortex** | **ODI_rh_cortex** | **ODI_lh_wm** | **ODI_rh_wm** | **ICVF_lh_cortex** | **ICVF_rh_cortex** | **ICVF_lh_wm** | **ICVF_rh_wm** | **Label** |
| --- | --- | --- | --- | --- | --- | --- | --- | --- |
| **0.2426** | 0.2269 | 0.2192 | 247 | 0.3525 | 0.3443 | 0.5265 | 0.5349 | TLE |
| **0.2696** | 0.2735 | 0.2207 | 233 | 408 | 0.4128 | 0.5702 | 0.5803 | TLE |
| **0.3639** | 0.3793 | 0.3213 | 0.3261 | 508 | 0.5322 | 0.6183 | 0.6324 | TLE |
| **0.2471** | 0.2503 | 0.2254 | 0.2149 | 0.3789 | 0.4137 | 0.5707 | 0.5915 | TLE |
| **0.2619** | 0.2543 | 0.2132 | 0.2062 | 359 | 0.3691 | 0.4875 | 0.4991 | TLE |
| **0.2915** | 0.2997 | 0.2327 | 231 | 0.4672 | 0.4792 | 565 | 0.5593 | TLE |
| **0.2875** | 0.2737 | 0.2435 | 0.2891 | 0.4355 | 0.3932 | 0.6198 | 607 | TLE |
| **0.2928** | 0.2941 | 0.2557 | 0.2619 | 0.3723 | 0.3811 | 0.5832 | 0.6024 | TLE |
| **0.2621** | 0.2317 | 0.2234 | 0.2225 | 0.3484 | 0.3267 | 0.5515 | 0.5396 | TLE |
| **253** | 0.2525 | 0.2345 | 0.2411 | 0.3731 | 0.3836 | 0.5302 | 0.5195 | TLE |
| **0.2594** | 0.2487 | 0.2424 | 0.2332 | 0.3694 | 0.3716 | 0.5633 | 578 | TLE |
| **0.2851** | 0.2825 | 0.2186 | 0.2039 | 0.4288 | 0.4307 | 0.5371 | 0.5482 | TLE |
| **0.2534** | 0.2497 | 0.2399 | 236 | 0.4157 | 404 | 0.5447 | 0.5501 | TLE |
| **0.2511** | 0.2422 | 0.2558 | 0.2522 | 0.4104 | 0.4344 | 0.5637 | 0.5706 | TLE |
| **0.2837** | 257 | 0.2283 | 0.2267 | 0.3883 | 0.3686 | 0.5435 | 574 | TLE |
| **0.2664** | 0.2483 | 0.2495 | 0.24 | 0.4087 | 0.4167 | 0.5451 | 0.5601 | TLE |
| **0.3252** | 0.3401 | 0.2653 | 0.2489 | 0.4877 | 0.4846 | 0.5693 | 0.5848 | TLE |
| **0.2215** | 0.2149 | 0.1952 | 0.2047 | 0.3444 | 0.3358 | 0.4563 | 0.4801 | TLE |
| **0.2843** | 0.2679 | 0.2394 | 0.2096 | 0.4531 | 0.4347 | 0.5911 | 0.5806 | TLE |
| **236** | 0.2347 | 0.2365 | 218 | 0.2916 | 0.2971 | 0.4985 | 0.5289 | TLE |
| **0.2358** | 0.2673 | 0.1895 | 0.2082 | 0.3782 | 0.4257 | 0.5083 | 507 | TLE |
| **0.2853** | 0.2849 | 0.2655 | 0.2449 | 0.4392 | 0.4639 | 0.5982 | 0.6153 | TLE |
| **0.2402** | 0.2339 | 0.2269 | 0.2265 | 0.3902 | 0.4093 | 0.5043 | 0.5225 | TLE |
| **0.2635** | 0.2664 | 0.2324 | 0.2247 | 0.3967 | 0.4055 | 0.5408 | 0.5463 | TLE |
| **281** | 0.2697 | 0.2377 | 0.2453 | 0.4802 | 0.4723 | 0.5652 | 0.5869 | TLE |
| **0.2343** | 0.2229 | 0.2153 | 0.2093 | 0.2987 | 0.3317 | 525 | 0.5468 | TLE |
| **248** | 0.2604 | 0.2354 | 0.2071 | 0.3138 | 0.3346 | 0.5436 | 0.5685 | TLE |
| **0.2502** | 0.2432 | 0.2148 | 0.2235 | 0.3101 | 0.3182 | 483 | 0.4492 | TLE |
| **246** | 0.2411 | 0.2165 | 0.2207 | 0.3591 | 0.3616 | 0.4939 | 0.5128 | TLE |
| **0.2989** | 0.2792 | 0.2323 | 0.2439 | 0.4306 | 0.4262 | 0.5195 | 553 | TLE |
| **0.2243** | 0.2316 | 0.2544 | 0.2625 | 0.33 | 334 | 0.4797 | 0.5055 | TLE |
| **0.2341** | 0.2352 | 0.2342 | 0.1877 | 0.3901 | 0.4103 | 0.5579 | 0.4909 | TLE |
| **0.2979** | 0.2792 | 0.2536 | 0.2394 | 0.4631 | 0.4604 | 0.5818 | 0.6013 | TLE |
| **0.2859** | 0.2952 | 0.2528 | 0.2469 | 0.4568 | 0.4801 | 0.6253 | 636 | TLE |
| **0.2802** | 0.2704 | 0.2319 | 0.2113 | 0.4448 | 0.4359 | 0.5667 | 0.5889 | TLE |
| **0.3404** | 0.3428 | 0.2774 | 0.2454 | 0.4447 | 0.4616 | 565 | 0.5695 | TLE |
| **0.2943** | 0.2656 | 0.2223 | 0.2208 | 406 | 0.3967 | 0.5303 | 0.5385 | TLE |
| **0.1046** | 0.1068 | 0.2428 | 0.1997 | 0.3044 | 0.3117 | 0.4479 | 0.3759 | TLE |
| **0.5207** | 0.5239 | 0.4623 | 0.4577 | 0.7335 | 0.7422 | 0.9059 | 0.9288 | TLE |
| **0.2969** | 0.3039 | 0.2632 | 0.26 | 0.4869 | 0.4951 | 0.6991 | 0.6898 | TLE |
| **0.3079** | 0.3099 | 0.2295 | 0.2485 | 0.4393 | 0.4294 | 0.5909 | 0.5915 | TLE |
| **0.2497** | 0.2658 | 0.2537 | 0.2415 | 0.3836 | 0.4153 | 0.5141 | 539 | TLE |
| **0.2423** | 237 | 0.2055 | 0.2069 | 0.3134 | 0.3261 | 0.4723 | 0.5007 | TLE |
| **0.2184** | 0.2133 | 0.2418 | 0.2295 | 0.3359 | 0.3478 | 559 | 517 | TLE |
| **0.2332** | 0.2213 | 0.2436 | 0.2345 | 0.3301 | 0.3372 | 0.5193 | 0.5314 | TLE |
| **0.2504** | 0.2529 | 0.2459 | 0.2388 | 0.34 | 0.3496 | 0.5227 | 0.5131 | TLE |
| **0.3895** | 0.3214 | 377 | 0.2895 | 0.5045 | 0.4713 | 0.6702 | 651 | TLE |
| **0.4691** | 0.4753 | 0.3595 | 0.3586 | 0.7839 | 0.8077 | 0.9265 | 0.9326 | TLE |
| **0.2539** | 0.2428 | 0.2394 | 239 | 0.3112 | 0.3061 | 0.5211 | 538 | TLE |
| **0.2279** | 0.2268 | 0.2581 | 0.2606 | 0.4388 | 0.4321 | 0.6097 | 0.6436 | TLE |
| **261** | 0.2565 | 0.2208 | 221 | 0.3865 | 0.3832 | 0.5783 | 0.5701 | TLE |
| **0.2964** | 0.3126 | 0.2321 | 0.2361 | 0.4634 | 0.4787 | 0.5835 | 0.5948 | TLE |
| **0.2516** | 0.2356 | 0.2384 | 0.2164 | 0.4149 | 0.4045 | 554 | 0.5477 | TLE |
| **0.2606** | 0.2486 | 0.2329 | 0.2212 | 0.3187 | 0.3308 | 0.5116 | 0.5372 | TLE |
| **0.4334** | 0.4358 | 0.3569 | 0.3758 | 733 | 0.7393 | 887 | 0.9329 | TLE |
| **392** | 0.2972 | 0.3439 | 255 | 0.4702 | 0.4001 | 0.6197 | 0.5973 | TLE |
| **0.3474** | 0.3426 | 0.2543 | 0.2416 | 467 | 0.4632 | 0.5803 | 0.6044 | TLE |
| **0.2192** | 0.2285 | 0.2116 | 0.2024 | 0.3359 | 0.3505 | 0.5061 | 0.5305 | TLE |
| **0.3342** | 0.3394 | 0.3394 | 0.3492 | 0.5751 | 0.5448 | 0.8188 | 0.8438 | TLE |
| **0.2765** | 0.2655 | 0.2559 | 0.2635 | 0.4494 | 0.4245 | 0.5931 | 0.5769 | TLE |
| **0.2366** | 0.2374 | 0.1919 | 0.1804 | 0.3359 | 0.3326 | 0.4279 | 0.4063 | TLE |
| **0.2257** | 211 | 0.2447 | 0.2326 | 0.3564 | 0.3287 | 0.5279 | 0.5614 | TLE |
| **0.2546** | 0.2678 | 0.2243 | 0.2104 | 0.3948 | 0.4163 | 0.5413 | 516 | TLE |
| **0.2492** | 262 | 0.2017 | 0.2168 | 0.3736 | 0.3731 | 0.4938 | 535 | TLE |
| **0.2675** | 0.2672 | 0.2199 | 0.2151 | 0.1886 | 0.2011 | 392 | 0.4244 | Keto_pre |
| **0.2745** | 0.2707 | 0.2085 | 0.2167 | 0.1847 | 0.2145 | 0.4301 | 0.4925 | Keto_post |

*Table S1:*

*Orientation dispersion index (ODI) and intracellular volume fraction (ICVF) for cerebellar cortex (left and right hemisphere) and cerebellar white matter (left and right hemisphere) before and after 3 months of ketogenic intermittent fasting (keto_pre/T0.1 and keto_post/T1.2) compared with a cohort of 64 patients with temporal lobe epilepsy (TLE)*
